# Serum bilirubin and the risk of chronic obstructive pulmonary disease exacerbations

**DOI:** 10.1186/s12931-017-0664-0

**Published:** 2017-10-24

**Authors:** Kirstin E. Brown, Don D. Sin, Helen Voelker, John E. Connett, Dennis E. Niewoehner, Ken M. Kunisaki, for the COPD Clinical Research Network

**Affiliations:** 1 Minneapolis VA Health Care System, Pulmonary, Critical Care, and Sleep Apnea (111N), One Veterans Drive, Minneapolis, MN 55417 USA; 2 University of Minnesota, Minneapolis, MN USA; 3 University of British Columbia, Vancouver, BC Canada

**Keywords:** Bilirubin, Biomarker, Pulmonary disease, Chronic obstructive

## Abstract

**Background:**

Bilirubin is a potent anti-oxidant and higher serum concentrations of bilirubin have been associated with better lung function, slower lung function decline, and lower incidence of chronic obstructive pulmonary disease (COPD). We sought to determine whether elevated bilirubin blood concentrations are associated with lower risk for acute exacerbations of COPD (AECOPD).

**Methods:**

We performed a secondary analyses of data in the Simvastatin for Prevention of Exacerbations in Moderate-to-Severe COPD (STATCOPE) and the Azithromycin for Prevention of Exacerbations of COPD (MACRO) studies. We used time-dependent multivariable Cox proportional hazards analyses, using bilirubin concentrations prior to first AECOPD as the exposure variable and time to first AECOPD as the outcome variable. STATCOPE was used for model development, with validation in MACRO.

**Results:**

In STATCOPE (*n* = 853), higher bilirubin was associated with a lower but statistically insignificant hazard for AECOPD, (adjusted hazard ratio [aHR] 0.89 per log_10_ increase [95%CI: 0.74 to 1.09; *p* = 0.26]). In the validation MACRO study (*n* = 1018), higher bilirubin was associated with a significantly lower hazard for AECOPD (aHR 0.80 per log_10_ increase [95%CI: 0.67 to 0.94; *p* = 0.008]).

**Conclusions:**

Bilirubin may be a biomarker of AECOPD risk and may be a novel therapeutic target to reduce AECOPD risk.

**Trial registrations:**

ClinicalTrials.gov NCT01061671 (registered 02 February 2010) and ClinicalTrials.gov NCT00325897 (registered 12 May 2006).

## Background

Acute exacerbations of chronic obstructive pulmonary disease (AECOPD) are associated with accelerated lung function decline, lower quality of life, increased mortality, and higher healthcare costs [1–3]. AECOPD pathogenesis is complex, but oxidative stress is commonly observed in AECOPD.

Anti-oxidant interventions such as carbocysteine and N-acetylcysteine have been shown to reduce risk for AECOPD in some [4, 5] but not all [6] trials. These interventions are targeted at increasing intracellular and extracellular concentrations of glutathione, a major endogenous antioxidant. Bilirubin is another potent antioxidant that protects lipids against oxidant stress and inhibits membrane-bound nicotinamide adenine dinucleotide phosphate oxidase, which is a large intracellular source of reactive oxygen species [7–9].

These antioxidant mechanisms may help explain why several large observational studies have shown that higher serum bilirubin concentrations are associated with better lung function [10], slower rate of FEV_1_ decline over 3 to 9 years [11] and a lower incidence of COPD, lung cancer, and all-cause mortality [12]. Together, these data suggest that higher serum bilirubin concentrations are associated with a lower risk of incident COPD and lower rates of COPD disease progression. No studies have investigated the association between serum bilirubin concentrations and AECOPD.

Given the important role of oxidant stress in AECOPD pathogenesis, we hypothesized that higher serum concentrations of bilirubin would be associated with a lower risk of AECOPD. We tested this hypothesis using data from two large multi-center cohorts of patients with COPD at high risk of AECOPD.

## Methods

We performed secondary analyses of data in the Simvastatin for the Prevention of Exacerbations in Moderate-to-Severe COPD (STATCOPE; ClinicalTrials.gov NCT01061671) and the Macrolide Azithromycin to Prevent Rapid Worsening of Symptoms Associated With Chronic Obstructive Pulmonary Disease (MACRO; ClinicalTrials.gov NCT00325897) studies.

### Study participants

STATCOPE and MACRO were designed as multi-center randomized controlled trials to test the efficacy of daily simvastatin (STATCOPE) and daily azithromycin (MACRO) to reduce the risk of acute exacerbations of COPD (AECOPD). The COPD Clinical Research Network conducted both studies with funding from the US National Heart, Lung and Blood Institute and Canadian Institutes of Health Research (for STATCOPE). The studies were approved by each participating center’s institutional review board and all study participants provided informed consent for study participation.

### Study participants

The design and results of both studies have been published [13, 14]. Inclusion criteria in both studies included age ≥ 40 years, post-bronchodilator forced expiratory volume in one second (FEV_1_)/forced vital capacity (FVC) <70%, FEV_1_ < 80% of predicted, ≥10 pack-year smoking history, and an increased risk of AECOPD (defined as home oxygen use, systemic corticosteroid or antibiotic usage for AECOPD, or having an emergency department visit or hospitalization for AECOPD in the year prior to study entry). Exclusion criteria included alcoholism and active liver disease (defined as transaminase elevations >1.5 times the upper limit of normal in STATCOPE, and >3 times the upper limit of normal in MACRO). STATCOPE additionally excluded those already treated with statins, those with indications to be on a statin according to the Adult Treatment Panel III risk stratification, and those with contraindications to statins. MACRO additionally excluded those with asthma, a resting heart rate greater than 100 beats per minute, a prolonged corrected QT (QTc) interval (>450 msec), the use of QT-prolonging medications, or hearing impairment.

STATCOPE was performed at 45 sites, and study follow-up time ranged from 21 to 1263 days, with a median (interquartile range) of 635 (329 to 990) days. The wide range of follow-up time in STATCOPE was largely due to the recommendation by the Data Safety and Monitoring Board for early termination of the trial, at a time when participants were still being actively recruited into the study. In contrast, MACRO continued to its planned closure date and had a follow-up time that ranged from 0 to 380 days, with a median of 200 (IQR 60 to 357) days. MACRO was performed at 17 sites.

### Data collection

AECOPD was defined identically in both studies as a complex of respiratory symptoms (increased or new onset) of more than one of the following: cough, sputum, wheezing, dyspnea, or chest tightness with a duration of at least three days requiring treatment with antibiotics or systemic steroids. Study personnel assessed AECOPD status monthly via clinic visits or telephone contacts.

Bilirubin concentrations were measured at baseline in both studies. In STATCOPE follow-up bilirubin was measured at months 6, 12, 18, and 24. In MACRO, follow-up bilirubin was measured more frequently, at months 1, 3, 6, 9, and 12.

### Statistical methods

We first utilized STATCOPE to develop and calibrate our analytic model and then validated the model using data from MACRO. The rationale for this ordering was due to several limitations of the STATCOPE cohort, compared to the MACRO cohort for this analysis, including STATCOPE’s smaller sample size, variable follow-up times, and less frequent bilirubin measurements (every 6 months in STATCOPE vs. every 1–3 months in MACRO).

We included all study participants who had at least one bilirubin measurement and at least one follow-up contact. One hundred five participants were in both STATCOPE and MACRO; these participants were included in the STATCOPE development/calibration dataset and were then excluded from the MACRO validation analysis.

We used time-dependent multivariable Cox proportional hazards analyses, using log_10_-transformed bilirubin concentrations prior to first AECOPD as the exposure variable of interest and time to first AECOPD as the outcome variable of interest. In contrast to traditional (i.e. non-time dependent) Cox models using only a static measure of exposure, time-dependent models account for intrinsically variable exposures that might influence outcomes (in our case serum bilirubin that has been shown to vary between several visits over a year [15]). Time-dependent models also can be more robust than static models, as they utilize all available data. Due to the availability of repeated bilirubin measurements in both of our cohorts, we elected to use this time-dependent approach.

Our development and calibration model included analyses of several clinical variables treated as continuous or categorical data, along with interaction terms. Covariates were those previously shown to affect AECOPD risk including age, sex, race, body mass index (BMI), chronic bronchitis, respiratory health status (as assessed by the St. George’s Respiratory Questionnaire [SGRQ] score), ethanol consumption, smoking status, post-bronchodilator FEV_1_%predicted, inhaled medications, use of supplemental oxygen, hospitalization or emergency department visit within the previous year, and steroid or antibiotic use within the previous year. Although simvastatin was shown to not affect AECOPD risk, we felt it important to also include treatment assignment (i.e. simvastatin vs. placebo) into the initial full development model. Covariates from this full STATCOPE development model were included in a final reduced STATCOPE model if they were significant at *p* < 0.10 by backwards stepwise regression; we forced treatment assignment into the reduced model also. We did not access the MACRO dataset until after the final reduced STATCOPE model was agreed upon.

In the better-powered MACRO validation analysis, we applied the final STATCOPE model, and we forced treatment assignment (i.e. azithromycin vs. placebo) into the model, due to azithromycin’s proven effect in reducing AECOPD risk and unclear effects on bilirubin concentrations [14].

Statistical analyses were conducted using SAS 9.3 (SAS Institute, Cary, NC, USA).

## Results

Among the 885 participants enrolled in STATCOPE, 853 had bilirubin concentrations measured and had at least one follow-up contact for determination of AECOPD status. These 853 participants formed the development/calibration sample for this study. Mean age of these STATCOPE participants was 62 years, 56% were male, 30% were current smokers, mean FEV_1_ was 1.19 L (42% of predicted), and 51% had been to an emergency department or hospitalized for AECOPD within the year prior to enrollment. Mean bilirubin concentrations were between 0.62–0.65 mg/dL, which is well within the general population normal range of 0.20–1.20 mg/dL. Additional baseline characteristics of these STATCOPE participants are presented in Table 1.

**Table 1:** Participant characteristics in development/calibration dataset (STATCOPE = Simvastatin for the Prevention of Exacerbations in Moderate-to-Severe COPD) and validation dataset (MACRO = Macrolide Azithromycin to Prevent Rapid Worsening of Symptoms Associated With Chronic Obstructive Pulmonary Disease)

	STATCOPE (*n* = 853)	MACRO (*n* = 1018)
Age, years	62 ± 8	66 ± 9
Male sex - no. (%)	479 (56%)	606 (60%)
Race - no. (%)		
Black	177 (21%)	138 (14%)
White	651 (77%)	830 (82%)
Other	20 (2%)	50 (5%)
BMI, kg/m^2^	27.2 ± 6.9	27.8 ± 6.2
Alcohol consumption, drinks per day	0.48 ± 0.85	0.40 ± 1.03
Currently smoking - no. (%)	257 (30%)	222 (22%)
Oxygen use - no. (%)	408 (48%)	604 (59%)
ED visit or hospitalized for AECOPD in year prior to enrollment - no. (%)	434 (51%)	517 (51%)
Steroids or antibiotics in previous year - no. (%)	715 (84%)	865 (85%)
FEV_1_/FVC ratio, %	44 ± 13	43 ± 13
Post-bronchodilator FEV_1_, L	1.19 ± 0.57	1.12 ± 0.51
Post-bronchodilator FEV_1_, % predicted	42 ± 18	40 ± 16
GOLD category - no. (%)		
1	–	1 (0.1%)
2	278 (33%)	277 (27%)
3	293 (35%)	410 (40%)
4	277 (33%)	327 (32%)
Mean Serum Bilirubin (mg/dL)		
Enrollment/baseline	0.65 ± 0.30	0.64 ± 0.29
Month 1	–	0.63 ± 0.28
Month 3	–	0.63 ± 0.28
Month 6	0.62 ± 0.28	0.63 ± 0.27
Month 9	–	0.63 ± 0.28
Month 12	0.65 ± 0.28	0.64 ± 0.28
Month 18	0.63 ± 0.27	–
Month 24	0.65 ± 0.28	–

*Abbreviations*: *AECOPD* acute exacerbation of chronic obstructive pulmonary disease, *BMI* body mass index, *ED* emergency department, *FEV*_*1*_ forced expiratory volume in one second, *FVC* forced vital capacity, *GOLD* Global Initiative for Chronic Obstructive Lung Disease

Of these STATCOPE participants, 475 (56%) experienced an AECOPD during the study. Covariates that were independently associated (at *p* < 0.10) with time to first AECOPD included male sex, black race, BMI, chronic bronchitis, supplemental oxygen use, St. George’s Respiratory Questionnaire (SGRQ) score, inhaler use, and steroid or antibiotic use within the past one year (Table 2). Covariates that were not independently associated with time to AECOPD included age, hospitalization for AECOPD within the previous year, ethanol use, FEV_1_% predicted, and current smoking. Follow-up bilirubin concentrations were not different between those assigned to simvastatin vs. placebo.

**Table 2:** Full STATCOPE development model. Time-dependent multivariable Cox proportional hazards analyses for risk of acute exacerbation of chronic obstructive pulmonary disease (AECOPD) and bilirubin prior to first AECOPD

Parameter	aHR	95% CI	*p*-value
STATCOPE Full Development Cohort Model			
Treatment assignment	0.93	0.77–1.11	0.41
Age	1.00	0.99 to 1.01	0.69
Male sex	0.84	0.69–1.01	0.07
Black race	0.66	0.51–0.86	0.002
BMI, kg/m^2^	0.99	0.97–1.00	0.07
Chronic bronchitis	1.22	1.00–1.49	0.05
Supplemental oxygen use	1.26	1.02–1.58	0.04
SGRQ score	1.02	1.01–1.02	<0.001
Inhaler use^a^ - none (0 of 3 classes: LABA, LAMA, ICS)	0.57	0.39–0.84	0.02
Inhaler use^a^ - 1 of 3 classes	0.96	0.72–1.29	0.10
Inhaler use^a^ - 2 of 3 classes	0.74	0.59–0.93	0.40
Steroid or antibiotic use for AECOPD in year prior to enrollment	1.52	1.13–2.05	0.006
Hospitalized for AECOPD in year prior to enrollment	1.13	0.92–1.39	0.25
Ethanol use, drinks/day	0.99	0.88–1.10	0.79
FEV_1_, % predicted	1.00	0.99–1.00	0.19
Current smoker	0.84	0.66–1.05	0.13
**BILIRUBIN (per log**_**10**_ ** increase)**	**0.90**	**0.74–1.09**	**0.28**

*Abbreviations*: *AECOPD* acute exacerbation of chronic obstructive pulmonary disease, *BMI* body mass index, *ED* emergency department, *FEV*_*1*_ forced expiratory volume in one second, *ICS* inhaled corticosteroid, *LABA* long-acting beta agonist, *LAMA* long-acting antimuscarinic, *SGRQ* St. George’s Respiratory Questionnaire

^a^Referent group is 3-class inhaler therapy (long-acting beta agonist, long-acting antimuscarinic, and inhaled corticosteroid)

Bilirubin is presented in bold, as this was the primary predictor variable

In the STATCOPE development dataset, higher bilirubin concentrations were independently associated with a lower but statistically insignificant hazard ratio for AECOPD (adjusted hazard ratio [aHR] in the final reduced model of 0.89 per log_10_ increase in bilirubin [95%CI: 0.74 to 1.09; *p* = 0.26) (Table 3).

**Table 3:** Reduced STATCOPE development cohort model. Time-dependent multivariable Cox proportional hazards analyses for risk of acute exacerbation of chronic obstructive pulmonary disease (AECOPD) and bilirubin prior to first AECOPD

Parameter	aHR	95% CI	*p*-value
STATCOPE Reduced Development Cohort Model			
Treatment assignment	0.92	0.77–1.11	0.38
Male sex	0.86	0.72–1.04	0.12
Black race	0.66	0.51–0.84	<0.001
BMI (kg/m^2^)	0.98	0.97–1.00	0.02
Chronic bronchitis	1.17	0.97–1.42	0.11
Supplemental oxygen use	1.37	1.12–1.67	0.002
SGRQ score	1.02	1.01–1.02	<0.001
Inhaler use^a^ - none (0 of 3 classes: LABA, LAMA, ICS)	0.52	0.36–0.76	0.007
Inhaler use^a^ - 1 of 3 classes	0.91	0.68–1.22	0.12
Inhaler use^a^ - 2 of 3 classes	0.72	0.58–0.90	0.51
Steroid or antibiotic use in year prior to enrollment	1.62	1.22–2.16	0.001
**BILIRUBIN (per log**_**10**_ ** increase)**	**0.89**	**0.74–1.09**	**0.26**

*Abbreviations*: *BMI* body mass index, *ICS* inhaled corticosteroid, *LABA* long-acting beta agonist, *LAMA* long-acting antimuscarinic, *SGRQ* St. George’s Respiratory Questionnaire

^a^Referent group is 3-class inhaler therapy (long-acting beta agonist, long-acting antimuscarinic, and inhaled corticosteroid)

Bilirubin is presented in bold, as this was the primary predictor variable

This final model was then applied to the larger, better-powered MACRO validation cohort. Of the 1142 MACRO participants, 1024 did not participate in STATCOPE. 1018 of the 1024 participants had a measured bilirubin concentration with at least one follow-up contact for AECOPD status determination and this comprised the validation cohort. MACRO participants were similar to STATCOPE participants, with a mean age of 66 years, 60% were male, 22% were current smokers, and mean FEV_1_ was 1.12 L (40% of predicted) (Table 1). 640 (63%) of MACRO participants experienced an AECOPD during the study. Follow-up bilirubin concentrations were not different between those assigned to azithromycin vs. placebo. In this validation dataset, higher bilirubin concentrations were independently associated with a statistically significantly lower hazard ratio for AECOPD. The point estimate was similar to that observed in STATCOPE, but with a narrower confidence interval (aHR 0.80 per log_10_ increase in bilirubin [95%CI: 0.67 to 0.94; *p* = 0.008]) (Table 4).

**Table 4:** MACRO validation cohort model. Time-dependent multivariable Cox proportional hazards analyses for risk of acute exacerbation of chronic obstructive pulmonary disease (AECOPD) and bilirubin prior to first AECOPD

Parameter	aHR	95% CI	*p*-value
MACRO Validation Cohort Model			
Treatment assignment	0.73	0.63–0.86	<0.001
Male sex	0.78	0.66–0.91	0.002
Black race	1.01	0.80–1.27	0.95
BMI (kg/m^2^)	0.99	0.98–1.00	0.07
Chronic bronchitis	1.22	1.03–1.44	0.02
Supplemental oxygen use	1.29	1.09–1.53	0.003
SGRQ score	1.01	1.00–1.01	0.003
Inhaler use^a^ - none (0 of 3 classes: LABA, LAMA, ICS)	0.74	0.54–1.03	0.20
Inhaler use^a^ - 1 of 3 classes	0.84	0.66–1.07	0.71
Inhaler use^a^ - 2 of 3 classes	0.91	0.76–1.09	0.52
Steroid or antibiotic use in year prior to enrollment	1.64	1.28–2.10	<0.001
**BILIRUBIN (per log**_**10**_ ** increase)**	**0.80**	**0.67–0.94**	**0.008**

*Abbreviations*: *BMI* body mass index, *ICS* inhaled corticosteroid, *LABA* long-acting beta agonist, *LAMA* long-acting antimuscarinic, *SGRQ* St. George’s Respiratory Questionnaire

^a^Referent group is 3-class inhaler therapy (long-acting beta agonist, long-acting antimuscarinic, and inhaled corticosteroid)

Bilirubin is presented in bold, as this was the primary predictor variable

## Discussion

Our data lend support to the hypothesis that higher circulating bilirubin concentrations are associated with a lower risk of AECOPD. Our findings are consistent with other observational data suggesting clinically important cardiopulmonary health benefits associated with higher bilirubin concentrations.

Several studies in the cardiac literature have demonstrated that higher blood bilirubin concentrations are associated with a lower risk of cardiovascular disease including peripheral vascular disease, carotid intimal-medial thickness, and stroke [16]. A study in the Framingham Heart Study Offspring cohort showed that among 1780 individuals with 24 years of follow-up, those with higher bilirubin due to a genetic polymorphism affecting the UGT1A1 enzyme of bilirubin metabolism (the enzyme defect that leads to Gilbert’s syndrome) had approximately one-third the risk of cardiovascular events compared to wild-type carriers with normal bilirubin concentrations [17]. The significant relationship between genetically determined bilirubin and cardiovascular events (an example of a so-called ‘Mendellian randomization’ study design) provides some additional support to a causal relationship. However, a more recent meta-analysis did not support an association between genetically elevated bilirubin and reduced risk of ischemic heart disease [18], thus, the potential protective role of bilirubin in cardiovascular disease pathogenesis is not fully known.

In addition to the data suggesting a potential cardiac benefit to higher blood bilirubin concentrations, emerging data suggest pulmonary benefits as well. A cross-sectional, population-based spirometry study of 4195 smokers and non-smokers in Switzerland showed that elevated serum concentrations of bilirubin and a genetic polymorphism associated with higher bilirubin concentrations were both independently associated with better lung function [10].

These cross-sectional findings were supported by a subsequent longitudinal study of 4680 North American smokers aged 35 to 60 years old with mild to moderate COPD at study entry, where higher serum bilirubin at baseline was associated with higher baseline FEV_1_ and a significantly slower rate of FEV_1_ decline over 3 to 9 years of prospective follow-up [11]. In this cohort of persons with COPD, there was also an association between higher serum bilirubin and a lower risk of coronary heart disease mortality over 15 years.

The largest study to investigate the association between serum bilirubin and pulmonary outcomes was conducted using a UK primary care research database [12]. In this longitudinal sample of 504,206 adults with a median follow-up time of 8 years, higher serum bilirubin concentrations were associated with a significantly lower incidence of COPD (adjusted incidence rate ratio of 0.92 [0.89 to 0.95] per 0.1 mg/dL increase in bilirubin in men and 0.89 [0.86 to 0.93] in women]. Higher bilirubin was also associated with a lower incidence of lung cancer and all-cause mortality.

Together, these data suggest that higher bilirubin concentrations are associated with a lower risk of incident COPD and lower rates of disease progression. We extend these data by demonstrating that elevated serum bilirubin concentrations are associated with a lower risk of AECOPD, one of the most important clinical outcomes for patients with COPD.

Our study has several strengths, including the ability to perform analyses in two large, multi-center trials that used very similar entry criteria and carefully collected AECOPD data, the primary outcome of both original studies. Unlike many epidemiologic studies that conduct analyses within only a single cohort, we were uniquely able to develop our analytic model in an exploratory fashion in the STATCOPE dataset, knowing from the outset that this would be a somewhat underpowered cohort due to infrequent bilirubin measurements, smaller sample size, fewer exacerbations, and variable follow-up time. We were thus able to reserve the better-powered MACRO dataset for validation purposes only. Moreover, the serial measurements of serum bilirubin during the observational period, especially the frequent measures in the MACRO validation dataset, enabled us to use a dynamic and flexible time-dependent Cox model to determine the relationship between bilirubin and incident exacerbations.

Our study has some limitations, including its reliance on bilirubin assays performed in clinical laboratories at study sites, rather than in a central laboratory. However, bilirubin is a well-established assay with well-established standard operating procedures in clinical laboratories, and an inter-laboratory coefficient of variation of only 1% to 3% [19]. The study population was also limited to those from North America at high risk of AECOPD, so our data do not apply to those at lower risk of COPD and may not apply to patients with COPD in other regions of the world. Perhaps most importantly, our study is observational and therefore unable to prove causality. High bilirubin concentrations could potentially be confounded by other healthy behaviors associated with these pulmonary outcomes.

Our study was not designed to answer mechanistic questions regarding how bilirubin might confer pulmonary benefits, but biologic plausibility comes from publications demonstrating that bilirubin is a potent antioxidant [7]. Indeed, bilirubin may be the most potent in vivo antioxidant to protect lipids against oxidant stress, tissue degeneration and death [8]. Smoking and other environmental oxidant insults significantly reduce serum bilirubin concentrations, but shortly after smoking cessation, serum bilirubin rapidly increases [20]. A recent study using a rat model of COPD showed that exogenous administration of bilirubin (as a therapeutic agent) reduced lung and systemic inflammation, suppressed regional oxidative lipid damage and prevented progression of histological changes of emphysema [21]. Bilirubin also inhibits membrane-bound nicotinamide adenine dinucleotide phosphate oxidase, which is a large intracellular reactive oxygen species source [9].

In summary, cellular and animal model data suggest that higher concentrations of bilirubin provide antioxidant benefits to the lung, while observational human data support the notion that higher bilirubin concentrations are associated with better COPD-related outcomes. Our data are consistent with these observations and suggest that higher bilirubin concentrations are associated with a lower risk for AECOPD.

## Conclusions

Among individuals with moderate-to-severe COPD without active liver disease, higher serum bilirubin concentrations are independently associated with a lower risk for AECOPD. Bilirubin may be a novel biomarker of AECOPD risk and may represent a novel therapeutic target for future investigations.

## References

[1] Donaldson GC, Seemungal TAR, Bhowmik A, Wedzicha JA (2002). Relationship between exacerbation frequency and lung function decline in chronic obstructive pulmonary disease. Thorax.

[2] Seemungal TAR, Donaldson GC, Paul EA, Bestall JC, Jeffries DJ, Wedzicha JA (1998). Effect of exacerbation on quality of life in patients with chronic obstructive pulmonary disease. Am J Respir Crit Care Med.

[3] Schmidt SAJ, Johansen MB, Olsen M, Xu X, Parker JM, Molfino NA, Lash TL, Sørensen HT, Christiansen CF (2014). The impact of exacerbation frequency on mortality following acute exacerbations of COPD: a registry-based cohort study. BMJ Open.

[4] Zheng JP, Kang J, Huang SG, Chen P, Yao WZ, Yang L, Bai CX, Wang CZ, Wang C, Chen BY, Shi Y, Liu CT, Chen P, Li Q, Wang ZS, Huang YJ, Luo ZY, Chen FP, Yuan JZ, Yuan BT, Qian HP, Zhi RC, Zhong NS (2008). Effect of carbocisteine on acute exacerbation of chronic obstructive pulmonary disease (PEACE study): a randomised placebo-controlled study. Lancet.

[5] Zheng JP, Wen FQ, Bai CX, Wan HY, Kang J, Chen P, Yao WZ, Ma LJ, Li X, Raiteri L, Sardina M, Gao Y, Wang BS, Zhong NS, PANTHEON study group (2014). Twice daily N-acetylcysteine 600 mg for exacerbations of chronic obstructive pulmonary disease (PANTHEON): a randomised, double-blind placebo-controlled trial. Lancet Respir Med.

[6] Decramer M, Rutten-van Mölken M, Dekhuijzen PN, Troosters T, van Herwaarden C, Pellegrino R, van Schayck CP, Olivieri D, Del Donno M, De Backer W, Lankhorst I, Ardia A (2005). Effects of N-acetylcysteine on outcomes in chronic obstructive pulmonary disease (bronchitis randomized on NAC cost-utility study, BRONCUS): a randomised placebo-controlled trial. Lancet.

[7] Stocker R, Glazer AN, Ames BN (1987). Antioxidant activity of albumin-bound bilirubin. Proc Natl Acad Sci U S A.

[8] Sedlak TW, Saleh M, Higginson DS, Paul BD, Juluri KR, Snyder SH (2009). Bilirubin and glutathione have complementary antioxidant and cytoprotective roles. Proc Natl Acad Sci U S A.

[9] Brandes RP, Weissmann N, Schröder K (2010). NADPH oxidases in cardiovascular disease. Free Radic Biol Med.

[10] Curjuric I, Imboden M, Adam M, Bettschart RW, Gerbase MW, Künzli N, Rochat T, Rohrer L, Rothe TB, Schwartz J, Stolz D, Tschopp JM, von Eckardstein A, Kronenberg F, Probst-Hensch NM (2014). Serum bilirubin is associated with lung function in a Swiss general population sample. Eur Respir J.

[11] Apperley S, Park HY, Holmes DT, Man SFP, Tashkin D, Wise RA, Connett JE, Sin DD (2015). Serum bilirubin and disease progression in mild COPD. Chest.

[12] Horsfall LJ, Rait G, Walters K, Swallow DM, Pereira SP, Nazareth I, Petersen I (2011). Serum bilirubin and risk of respiratory disease and death. JAMA.

[13] Criner GJ, Connett JE, Aaron SD, Albert RK, Bailey WC, Casaburi R, Cooper JA, Curtis JL, Dransfield MT, Han MK, Make B, Marchetti N, Martinez FJ, Niewoehner DE, Scanlon PD, Sciurba FC, Scharf SM, Sin DD, Voelker H, Washko GR, Woodruff PG, Lazarus SC, COPD clinical research network; Canadian Institutes of Health Research (2014). Simvastatin for the prevention of exacerbations in moderate-to-severe COPD. N Engl J Med.

[14] Albert RK, Connett J, Bailey WC, Casaburi R, Cooper JA, Criner GJ, Curtis JL, Dransfield MT, Han MK, Lazarus SC, Make B, Marchetti N, Martinez FJ, Madinger NE, McEvoy C, Niewoehner DE, Porsasz J, Price CS, Reilly J, Scanlon PD, Sciurba FC, Scharf SM, Washko GR, Woodruff PG, Anthonisen NR, COPD Clinical Research Network (2011). Azithromycin for prevention of exacerbations of COPD. N Engl J Med.

[15] Nicolau GY, Haus E, Lakatua DJ, Bogdan C, Sackett-Lundeen L, Popescu M, Petrescu E, Robu E, Reilly C (1986). Circannual rhythms of laboratory parameters in serum of elderly subjects. Evaluation by cosinor analysis. Endocrinologie.

[16] Schwertner HA, Vitek L (2008). Gilbert syndrome, UGT1A1*28 allele, and cardiovascular disease risk: possible protective effects and therapeutic applications of bilirubin. Atherosclerosis.

[17] Lin J, O'Donnell CJ, Schwaiger JP, Cupples LA, Lingenhel A, Hunt SC, Yang S, Kronenberg F (2006). Association between the UGT1A1*28 allele, bilirubin levels, and coronary heart disease in the Framingham heart study. Circulation.

[18] Stender S, Frikke-Schmidt R, Nordestgaard BG, Grande P, Tybjaerg-Hansen A (2013). Genetically elevated bilirubin and risk of ischaemic heart disease: three Mendelian randomization studies and a meta-analysis. J Intern Med.

[19] Lo SF, Kytzia HJ, Schumann G, Swartzentruber M, Vader HL, Weber F, Doumas BT (2009). Interlaboratory comparison of the Doumas bilirubin reference method. Clin Biochem.

[20] O’Malley SS, Wu R, Mayne ST, Jatlow PI (2014). Smoking cessation is followed by increases in serum bilirubin, an exogenous antioxidant associated with lower risk of lung cancer and cardiovascular disease. Nicotine Tob Res.

[21] Wei J, Zhao H, Fan G, Li J (2015). Bilirubin treatment suppresses inflammation in a rat model of smoke-induced emphysema. Biochem Biophys Res Commun.

